# Acute Myocardial Infarction Caused by an Anomalous Right Coronary Artery Occlusion Presenting with Precordial ST Elevation

**DOI:** 10.1155/2017/3972830

**Published:** 2017-07-16

**Authors:** Bruno da Silva Matte, Alexandre Damiani Azmus

**Affiliations:** Hospital N.S. Conceição, Porto Alegre, RS, Brazil

## Abstract

Acute coronary syndrome with precordial ST segment elevation is usually related to left anterior descending artery occlusion, although isolated right ventricular infarction has been described as a cause of ST elevation in V1–V3 leads. We present a case of a patient with previous inferior wall infarction and new acute ST elevation myocardial infarction (STEMI) due to proximal right coronary thrombotic occlusion resulting in right ventricular infarction with precordial ST elevation and sinus node dysfunction. The patient was treated with successful rescue angioplasty achieving resolution of acute symptoms and electrocardiographic abnormalities.

## 1. Introduction

Several algorithms were established to locate the culprit vessel in ST elevation myocardial infarction (STEMI) with good accuracy [1–3], but in some clinical scenarios including previous myocardial infarction, it can be misleading if thorough electrocardiogram evaluation is not performed. We describe a case in which precordial ST elevation occurred in a patient with proximal right coronary artery acute occlusion and was treated with coronary angioplasty.

## 2. Case Presentation

A 42-year-old male patient with history of hypertension, dyslipidemia, diabetes mellitus, and previous myocardial infarction (MI) presented to the emergency department with acute coronary syndrome. His past coronary event was an inferior MI which was treated with thrombolysis six years ago within 7 hours of onset. Catheterization at that time revealed anomalous right coronary artery originating from the left Valsalva sinus with moderate proximal obstruction and the patient received medical treatment. He had recently performed a clinical evaluation with a normal treadmill test and normal echocardiogram in another hospital.

**Figure 1:**
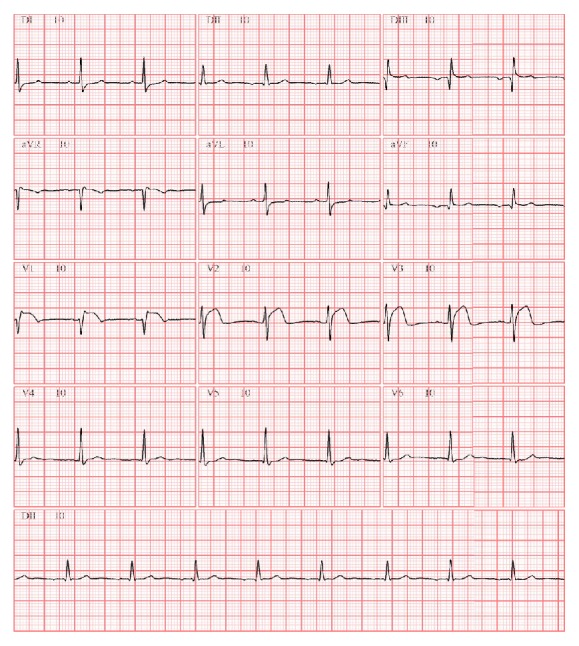
Admission electrocardiogram.

**Figure 2:**
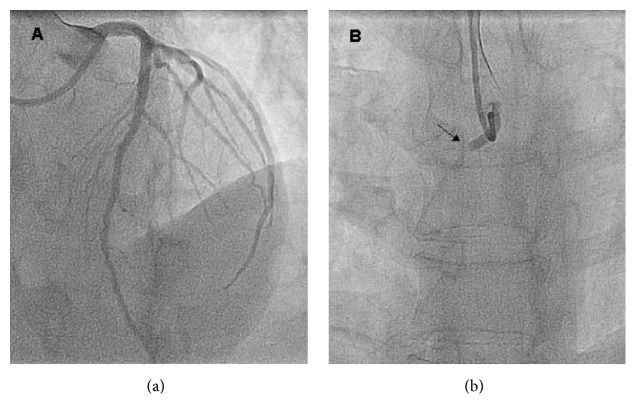
Coronary angiography. (a) Normal left coronary artery; (b) proximal occlusion of anomalous right coronary artery (black arrow) originating in the left Valsalva sinus.

**Figure 3:**
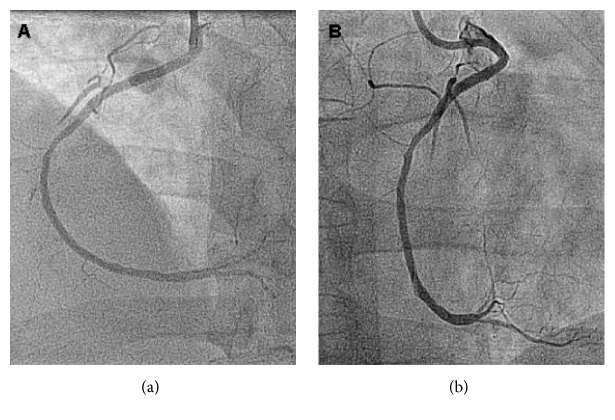
Post-PCI angiogram. RCA seen in both LAO (a) and RAO (b) views. Good poststenting result in proximal RCA (TIMI 3) but slow flow (TIMI 2) in RV marginal branches. RCA: right coronary artery; LAO: left anterior oblique; RAO: right anterior oblique; TIMI: thrombolysis in myocardial infarction; RV: right ventricular.

He presented to the emergency department of a non-24/7 primary PCI medical centre with acute onset (2 hours) of severe chest pain and diaphoresis. On admission, he was anxious, his blood pressure was 80/50 mmHg, and he had normal heart and lung auscultation. His first electrocardiogram (Figure 1) showed ectopic atrial rhythm, inferior inactive zone, and ST elevation in precordial leads V1–V3 (there were no previous electrocardiogram tracings available for comparison). He was treated with IV hydration, acetylsalicylic acid 300 mg, clopidogrel 600 mg, and thrombolysis with Tenecteplase 40 mg with improvement of peripheral perfusion and blood pressure. However, due to persistent chest pain, emergent coronary angiography was performed four hours later. Left main and circumflex were free of significant obstruction and left anterior descending artery had a moderate systolic dynamic compression caused by intramyocardial bridging in the mid segment with normal TIMI flow (Figure 2). Right coronary artery had its anomalous origin close to the left coronary sinus and was occluded in the proximal segment (Figure 3).

Percutaneous coronary intervention was performed in the right coronary artery through right radial approach using Amplatz 1 (6 French) guiding catheter. He received 8,000 U of unfractioned heparin. The lesion was easily crossed with a Runthrough 0.014 guidewire and predilated with a 2,5 × 20 mm balloon with flow restoration. A Zotarolimus eluting stent (Endeavor) 2,75 × 24 mm was implanted at 12 atm and postdilated with a 3,0 mm balloon at 20 atm with clinical and angiographic success. At the end of the procedure, the patient was asymptomatic and ECG showed resolution of ST elevation.

**Figure 4:**
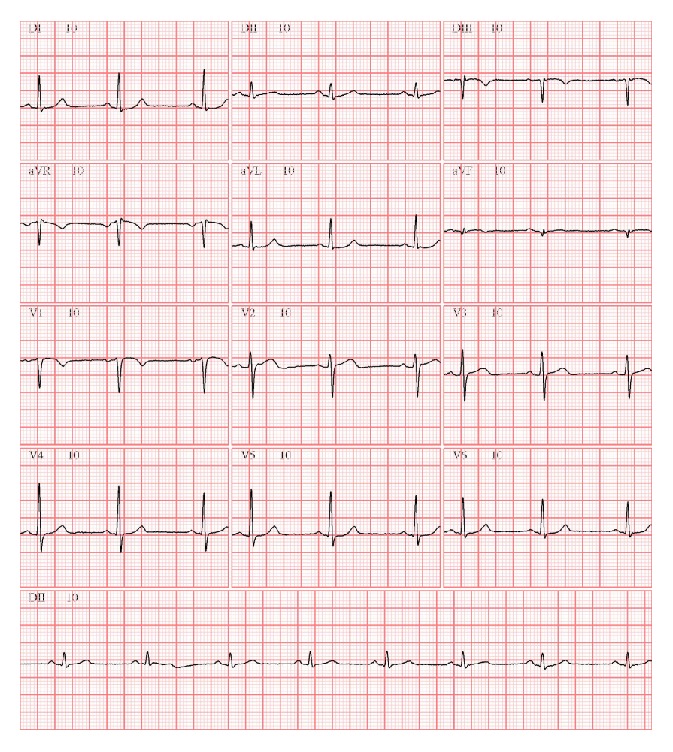
Predischarge electrocardiogram.

The patient had in-hospital course free of complications and predischarge evaluation revealed normal left and right ventricular function (ejection fraction 59%) and complete resolution of precordial electrocardiogram abnormalities and return to normal sinus rhythm (Figure 4). The patient was discharged six days after admission taking acetylsalicylic acid 100 mg, clopidogrel 75 mg, atorvastatin 80 mg, metoprolol 100 mg BID, and enalapril 10 mg BID. After an uneventful one-month follow-up, the patient was asymptomatic.

## 3. Discussion

Acute myocardial infarction (AMI) with precordial ST segment elevation is usually related to left anterior descending coronary artery thrombotic occlusion and is rarely seen in proximal right coronary occlusion [4]. Electrocardiogram is essential to locate the culprit vessel in AMI and extremely relevant for clinical reasons such as correct identification of AMI type and prognosis but also to guide the choice of devices in the catheterization laboratory. Although several algorithms were established to locate the culprit vessel with good results, their accuracy is compromised in some scenarios including previous myocardial infarction, coronary anomalies, previous coronary artery bypass surgery, left bundle block, and paced rhythms.

Some case reports counteract the classical electrocardiogram finding of ST elevation in DII, DIII, and AVF for right coronary acute occlusion, with most of them being the result of isolated right ventricular myocardial infarction (RVMI) when precordial ST elevation can occur [5–7]. Interestingly, Kanovsky el al. [8] reported in a survey of 300 patients with electrocardiographic defined RVMI (V4R ST segment elevation) that the culprit vessel was the right coronary artery in only 48% of the cases, the left anterior descending artery in 47%, and the circumflex artery in 5% of cases. One should also note that in this study RV function was not evaluated and we could only infer it to be worse in patients with RCA occlusion because of the greater magnitude of RV branches from this vessel. Zheng et al. [9] have selected some aspects that would suggest RCA occlusion/RVMI when precordial ST elevation is present: decreasing magnitude of ST segment elevation from V1 to V3, convex ST elevation, concomitant sinus node dysfunction, and V3-V4R ST elevation. Also, the authors suggest a complementary bedside evaluation with echocardiography in which RV dilation would add to the electrocardiogram approach.

In the reported case, the patient had a mild cardiogenic shock presentation due to STEMI with failed thrombolysis and clear angiographic definition of RCA as the culprit vessel, without significant stenosis on the left coronary artery. Rather important too, the RCA occlusion was proximal to the RV branches and “isolated” RVMI in the setting of a previous inferior wall myocardial infarction was considered the cause of the precordial ST elevation without ST elevation in leads DII, DIII, and AVF. Also noteworthy, sinus node dysfunction was present as ectopic atrial rhythm was shown at presentation and normalized to sinus rhythm only after reperfusion. Some of the above-mentioned electrocardiographic features were present such as convex ST elevation and sinus node dysfunction but decreasing ST elevation from V1 to V3 was not seen in this case. Unfortunately, because our institution only performs right precordial leads on admission in inferior myocardial infarctions, we do not have data on V3-V4R leads which we believe would be showing ST elevation.

## 4. Conclusion

Although uncommon in proximal RCA occlusion, the electrocardiographic pattern shown in this case may lead to erroneous culprit vessel definition and treatment delay in this severe clinical scenario. Thorough ECG evaluation and complementary quick bedside evaluation can improve diagnosis and shorten door-to-balloon time and result in better patient prognosis.
